# Effects of Resting Conditions on Tensile Properties of Acid Aggregate Hydraulic Asphalt Concrete

**DOI:** 10.3390/ma17143556

**Published:** 2024-07-18

**Authors:** Lei Bao, Min He, Shu Wang, Xinshuang Wu

**Affiliations:** 1 School of Civil Engineering and Architecture, Xi’an University of Technology, Xi’an 710048, China; lbao1013@163.com; 2 Power China Northwest Engineering Corporation Limited, Xi’an 710065, China; 13102213129@163.com (S.W.); wuxins@nwh.cn (X.W.); 3 State Key Laboratory of Eco-Hydraulics in Northwest Arid Region of China, Xi’an University of Technology, Xi’an 710048, China

**Keywords:** asphalt concrete, anti-stripping agent, constant temperature, tensile properties, adhesion, acid aggregates

## Abstract

**Highlights:**

**What are the main findings?**

- The effect of resting time on asphalt adhesion was most pronounced in comparison to temperature and spalling agent dosage.

**What is the implication of the main finding?**

- A combined micro- and macro-scale investigation was conducted to ascertain the impact of varying static conditions on the tensile properties of asphalt concrete.

**Abstract:**

This study addresses the issue of construction stagnation affecting the adhesion and tensile properties of hydraulic asphalt concrete with acid aggregate. It investigates the impact of rest periods on the tensile characteristics of such materials under standard construction conditions. The influence of varying rest durations and asphalt temperatures on the tensile behavior of the concrete is assessed through indoor experiments. The bonding between asphalt and aggregate is examined, along with the tensile property variations of the concrete. The study found that the standstill time significantly affects the adhesion of asphalt, with the adhesion decreasing progressively with increased temperature and rest time, irrespective of the addition of anti-stripping agents. However, the inclusion of these agents can mitigate the reduction in adhesion. Furthermore, the study identified that rest duration has a more substantial impact on adhesion than temperature. The splitting tests demonstrate that the tensile properties of asphalt concrete are considerably affected by the resting time. Over a period of 0, 10, 20, and 30 days of rest, an increase in splitting strength and a decrease in splitting displacement were observed. The findings offer valuable insights for predicting the tensile performance of asphalt concrete in practical engineering applications after a period of rest.

## 1. Introduction

Hydraulic asphalt concrete is mainly composed of aggregate and asphalt, with good impermeability and strong deformation ability [1,2]. In addition, the high economic benefits and the repair process of hydraulic asphalt concrete is relatively simple [3,4,5]. This material is dominant in hydraulic seepage control projects at home and abroad, and it is widely used in various seepage control structures, such as heart walls and pumped storage power station seepage control panels [6,7,8,9]. With the rapid development of water conservancy project construction, the number of projects involving hydraulic asphalt concrete is increasing, and the study of the mechanical properties of hydraulic asphalt concrete can not only avoid the instability and other safety problems of the seepage control structure of earth and stone dams, but it also has an important reference significance for the operation and maintenance of hydraulic seepage control projects.

The aggregate used for asphalt concrete can be differentiated into three categories according to the alkalinity modulus: acidic, alkaline, and neutral. Alkaline aggregate is more compatible with asphalt and has a high adhesion strength; therefore, in many construction projects, alkaline aggregate is preferred due to mechanical performance considerations. However, with the rapid development of China’s water conservancy projects, many projects have encountered difficulties in finding alkaline aggregate. Long-distance transport undoubtedly increases the cost of the project. The new water conservancy projects have been affected by the lack of high-quality alkaline aggregate materials, resulting in the prolonged duration of the phenomenon of the endless phenomenon [10]. Acidic aggregate is the most widely distributed, with the largest reserves. It has a high hardness, wear resistance, durability, and structural safety and stability. The embedded aggregate plays a role in this. However, due to its poor adhesion to asphalt, it is susceptible to asphalt spalling from the aggregate surface in the case of rainwater washout [11], which can subsequently lead to a reduction in the mechanical properties of asphalt concrete and structural stability, thereby increasing the likelihood of water damage and other common diseases [12]. It was demonstrated that the incorporation of an anti-spalling agent can enhance the adhesion between asphalt and acidic aggregate [13]. Some projects have attempted to utilize acidic aggregate, which employed the use of diorite acidic aggregate in situ [14]. This approach not only reduces the project cost and duration but also benefits the local area.

In the actual projects, asphalt is ready to use, but rain and snowstorms, water and power outages, mudslides, the construction of cultural relics, and other external factors would cause delays in the schedule, so configured asphalt needs to be static for the smooth progress of the project, and during this period, different static conditions, such as temperature, air, and radiation, can also have a certain impact on the performance of asphalt. Numerous scholars have made the following studies: Wang et al. [15] showed that the temperature has a large effect on the dynamic modulus of asphalt concrete. Pszczola [16] found that, at low temperatures, asphalt concrete with modifiers has a higher tensile capacity compared to asphalt concrete without modifiers. Wang et al. [17] also obtained the same conclusion in the study of mechanical properties of asphalt concrete at different temperatures. Gheibiet et al. [18] conducted a series of resonant column tests on asphalt concrete specimens in order to investigate the effect of temperature on the dynamic properties of asphalt concrete and obtained that temperature changes have a greater effect on shear wave velocity and shear modulus; for example, when the temperature increases from 0 °C to 22 °C, the shear modulus and the shear wave velocity decrease by about 25% and 13%, respectively. Li et al. [19] investigated the fracture resistance of 28 different asphalt mixtures at low temperatures. The fracture resistance was evaluated by performing semicircular bending fracture tests at three low temperatures. Two parameters, fracture energy and fracture toughness, were calculated. It was found that the asphalt content had a significant effect on the fracture energy but had little effect on the fracture toughness. Cheng et al. [20] explored the stiffness modulus of asphalt concrete, which was found to increase significantly at low temperatures. Islam et al. [21] found it to be susceptible to performance erosion during storage and transport in closed containers at temperatures above 160 °C. Ke [22] found that Styrene Ethylene Butylene Styrene (SEBS) is poorly compatible with asphalt and that modified asphalt undergoes severe phase separation during storage. Mohamed [23] found that additional aging and possible blending between Reclaimed Asphalt Pavement (RAP) and virgin binders take place inside the silo, which affects mixing properties. Li [24] measured the viscosity–temperature curves at different storage temperatures based on a Brinell viscometer, and the asphalt performed differently.

Compared with acidic aggregates, alkaline aggregates are preferred for mechanical properties in actual projects. However, with the rapid development of water conservancy project construction and many project construction areas around the situation of difficult-to-find alkaline aggregate, long-distance transport undoubtedly increases the cost of the project, including the new water conservancy project due to the lack of high-quality alkaline aggregate materials, which leads to the prolongation of the endless phenomenon [25]. The new water conservancy projects have been prolonged due to the lack of high-quality alkaline aggregate. Acidic aggregate has the widest distribution and the largest reserves, and it also has the characteristics of high hardness, strong wear resistance, and strong durability. Under embedded extrusion, the structural safety and stability between aggregates are quite good. However, because of its poor adhesion to asphalt, it is easy to cause asphalt to spill off from the aggregate surface after rainfall, which reduces the mechanical properties and structural stability of asphalt concrete and ultimately causes water damage and other common diseases [13]. It was found that the addition of an anti-stripping agent can reduce the mechanical properties and structural stability of asphalt concrete and enhance the adhesion between asphalt and acidic aggregate [26], which can not only reduce project costs but also shorten the construction period.

A large number of studies [27,28,29,30] showed that when earth and rock dam is subjected to stronger external loads, such as the excessive thickness of the overburden layer, or other site-specific conditions, which result in phenomena such as uneven settlement or the deflection of the dam foundation, areas such as the reverse-arc section of the hydraulic asphalt concrete panels and the structural intersections are subjected to different degrees of tensile stresses. In addition, as the height of the earth and rock dam increases, the peak tensile stress and its action area will likewise increase [13,31]. When the hydraulic asphalt concrete is under extreme external loading, the structure undergoes functional damage, and the stability and safety of the earth and rock dam will be greatly reduced [32,33]. Therefore, the tensile property is one of the most important indicators of the practical performance of asphalt concrete engineering.

Due to unforeseeable circumstances, the current project had to be halted. Consequently, the performance of the configured asphalt may change under varying static conditions. As a result, it is crucial to conduct research on the tensile properties of hydraulic asphalt concrete containing acidic aggregate under construction control factors such as different asphalt resting conditions. This paper investigated the effects of asphalt length and temperature on the tensile properties of hydraulic asphalt concrete with an acidic aggregate. Boiled and split tests were used to examine the adhesion between the asphalt and aggregate and the resulting changes in asphalt concrete tensile properties. The main research content focused on two aspects:

1. We studied the variation rule of adhesion between asphalt and acidic aggregate under different temperatures, resting times, and dosages of anti-stripping agents. We analyzed the effects of static length, temperature, type, and dosage of anti-stripping agent on the adhesion between asphalt and acidic aggregates.
2. We investigated the influence of resting conditions on the tensile properties of asphalt concrete. We conducted hydraulic asphalt concrete splitting tests under different static conditions such as 0, 10, 20, and 30 days and analyzed the changes in the tensile properties of asphalt concrete under different static conditions.

## 2. Materials and Test Methods

### 2.1. Raw Materials

#### 2.1.1. Asphalt Testing

The asphalt (modified I-C asphalt, Jingbo Petrochemical, Yimeng, China) was tested, and the results are shown in Table 1. The modified I-C asphalt of Jingbo Petrochemical was mixed with 0.8% SK-A anti-stripping agent to be used in the test, and the tested results are shown in Table 1 and Table 2. In this study, the asphalt concrete specimens were prepared and tested in accordance with the “Test Procedure for Hydraulic Asphalt Concrete” (DL/T 5362-2018) [34], which is a national standard of the People’s Republic of China. The preparation and testing methods were translated and adapted to align with the requirements of this research.

#### 2.1.2. Aggregate Testing

**(1)**  **Alkalinity modulus (*M*)**

The testing of the aggregates selected for this study was conducted in accordance with the Test Procedure for Hydraulic Asphalt Concrete (DL/T 5362-2018) [34]. The ratio (M) of CaO, MgO, FeO content to SiO_2_ in the chemical composition of the raw rock was calculated according to Equation (1):

(1) $$M=\frac{\mathrm{CaO}+\mathrm{MgO}+\mathrm{FeO}}{{\mathrm{SiO}}_{2}}$$

When ***M*** > 1, the aggregate is alkaline; when 0.6 < ***M*** < 1, the aggregate is neutral; and when ***M*** < 0.6, the aggregate is acidic.

**(2)**  **Mineral composition testing**

Table 3 presents the results of the chemical composition of the gneiss diorite (Gneiss Diorite), including the contents of SiO_2_ (Silicon dioxide), Al_2_O_3_ (Aluminum oxide), Fe_2_O_3_ (Iron(III) oxide), MgO (Magnesium oxide), and CaO (Calcium oxide), as well as the heat loss and the alkalinity modulus (***M***). A calculation can be made based on the data in the table to determine the value of ***M*** (Alkalinity modulus). The results of aggregate testing are shown in Table 3.

The selected material for this study was gneiss amphibolite, which was identified as an acidic rock with an alkalinity modulus ***M*** of 0.18. Aggregates with acidic characteristics are known to exhibit poor adhesion to asphalt, which may consequently affect the mechanical properties and durability of asphalt concrete.

#### 2.1.3. Coarse Aggregate

We selected the coarse aggregate (particle size of 19~2.36 mm) material to be fresh and hard, with no cracking. We looked for decomposition and other phenomena in the heating process, and we tested for acidity and strong adhesion with asphalt. Table 4 presents the criteria for the selection of coarse aggregates, including apparent density, adhesion to asphalt, content of needle and flake particles, crushing value, water absorption, mud clod content, and durability. The test results demonstrate that the selected coarse aggregate meets the technical requirements, exhibiting a high apparent density and satisfactory adhesion to asphalt. This is of paramount importance for guaranteeing the quality and performance of asphalt concrete. The materials used meet the technical requirements for coarse aggregate asphalt concrete panels.

#### 2.1.4. Fine Aggregates

We selected (particle size of 2.36–0.075 mm) aggregates with a hard and fresh texture, with no cracking, and that exhibited decomposition and other phenomena during the heating process. The good performance of the fine aggregate and the test results are shown in Table 5.

The fine aggregate obtained from the experiment had a hard and fresh texture and did not crack or decompose during the heating process, with good performance. However, the aggregates themselves were acidic, so measures should be taken to improve the adhesion between aggregates and asphalt.

#### 2.1.5. Fillers

The filler used in the test was acidic, and the related parameters were detailed in Table 6. It can be seen that the filler used in this test was acidic material, and the primary sources comprise aggregates and fillers extracted from natural ores and asphalt extracted from petroleum refining. These raw materials underwent processing and blending to fabricate asphalt concrete filler, which primarily serves as a surface layer for infrastructure. It facilitated excellent compression, abrasion, and water resistance. Furthermore, asphalt concrete filler may be utilized for surface paving in select industrial establishments and instances where waterproofing properties and abrasion resistance are necessary.

#### 2.1.6. Anti-Stripping Agents

The main raw materials of the asphalt (Shui Ke, Beijing, China) anti-stripping agent were rosin and other surfactants, which were solid particles at room temperature. The melting point was 60 °C, and they were easily soluble in asphalt. The boiling point of the anti-stripping agent was 230 °C, which had the advantage of stable performance at high temperatures and kept the adhesion basically undiminished for 30 days when the asphalt was sealed and stored at 130 °C. This anti-spalling agent has been developed in conjunction with water conservancy projects to meet the specific requirements of hydraulic asphalt concrete. Through the molecular structure design and synthesis of a new type of asphalt concrete spalling agent, the product has been shown to significantly improve the adhesion of asphalt and acidic aggregate, while maintaining the impact of other asphalt indicators. This class of anti-spalling agents exhibits excellent product performance, a broad range of adaptability, and good applicability.

#### 2.1.7. Grading

In this study, the content of each component of asphalt concrete was calculated based on the Ding Purong grading formula [35]. Asphalt concrete ratio selection was based on different grading indexes, filler dosages, and oil and stone compositions of different ratios, after the analysis of porosity, splitting strength and displacement, and other performance indicators were selected to ensure better performance of the ratio (porosity indicators can reflect the impermeability performance of asphalt concrete splitting tests; that is, the indirect tensile test can be relatively reflective of the strength and deformation properties of asphalt concrete).

Factors that affect the performance of asphalt concrete are as follows:

1. Properties of raw materials, including asphalt, coarse aggregates, fine aggregates, and fillers.
2. Mineral grading.
3. The amount of filler, i.e., the proportion of filler in the mineral.
4. Oil–rock ratio, i.e., the ratio of the mass of asphalt in the asphalt mixture to the mass of minerals in the asphalt mixture. Mineral gradation parameters include the maximum mineral particle size D_max_, gradation index r, or coarse and fine aggregate rate and filler F. Mineral gradation was determined using the theory of the maximum dense gradation, and the recommended formula was listed as follows:
  (2) $$Pi=F+(100-F)\frac{d_{i}^{r}-d_{0.075}^{r}}{D_{\max}^{r}-d_{0.075}^{r}}$$
  where *P_i_* is the passing rate of the sieve aperture, *F* is the amount of filler, with the particle size less than 0.075 mm, *D*_max_ is the maximum particle size of the mineral, mm, *d_i_* is a certain sieve size, *d*_0.075_ is the maximum particle size of the filler, and *r* is the grading index.

In this test, according to the previous engineering experience and experimental research results [36], the selected mix ratio was 7% of the oil and stone ratio, 9% of the filler dosage, and 0.5 of the grading index, and the detailed parameters are shown in Table 7.

### 2.2. Test Methods

#### 2.2.1. Boiling

**Figure 1:**
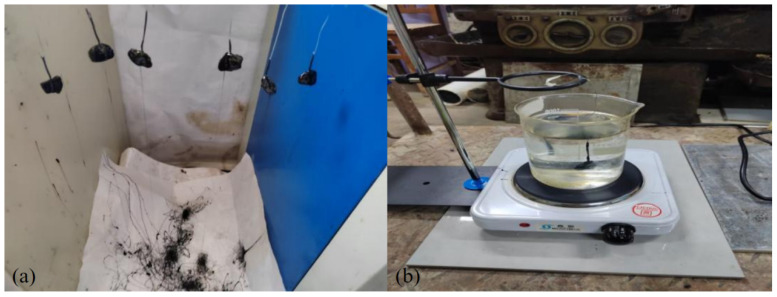
Boiling test diagram: (**a**) boiling test sample, (**b**) boiling test process.

The adhesion between asphalt doped with different contents of anti-stripping agents and gneissic amphibolite aggregate was tested according to the coarse aggregate and asphalt adhesion test (boiled water method) in the Test Procedure for Hydraulic Asphalt Concrete (DL/T 5362-2018). The study was conducted three times for each set of experiments, with the results averaged. If the three sets of experimental data values differed by more than 15 percent, the study was repeated. The test incorporated flaky amphibole as the selected aggregates, Jingbo’s modified asphalt, and two types of anti-stripping agents from the Shui Ke brand, the asphalt anti-stripping agent (SK-A, Beijing, China) and the new-generation non-ammonium asphalt anti-stripping agent (SA-100, Nanjing, China). The study focused on investigating the impact of various resting conditions, including resting time, temperature, and anti-stripping agent dosage, on the bond between the acidic aggregates and asphalt, using the water-boiling method. In the actual engineering construction, the mixing temperature of asphalt mixtures tended to change frequently. For instance, the mixing temperature of asphalt (No. 70, Chongqing, China) was about 160 °C, while that of modified asphalt was about 180 °C. Therefore, this test was conducted at the temperature range of 120 °C to 200 °C. In order to evaluate the effects of different external factors on the duration of the stoppage of work, the resting time for this test was set at 0–30 days. Generally, the amount of anti-peeling agent doping used in the project was 0. To facilitate the investigation of the impact of various resting conditions (resting time, temperature, anti-peeling agent dosage) on asphalt and acid aggregate adhesion, asphalt samples with values ranging between 2% and 0.5% were used. Similarly, to observe the effect of anti-stripping agents on asphalt and aggregate adhesion, the anti-stripping agent values used in the test were 0%, 0.4%, 0.6%, and 0.8%, respectively. The test procedure for the assessment of the adhesion of aggregates to asphalt by the boiled water method is as follows:

1. The aggregate particles should be selected to be free from sharp edges and with a shape that is as close to cubic as possible. They should then be cleaned and placed in an oven at a temperature of 105 °C ± 5 °C to dry the aggregate particles completely. Once this process is complete, the particles should be removed from the oven and allowed to cool to room temperature.
2. A fine wire should then be used to bake the clean aggregate particles in an oven at 105°C ± 5°C for one hour.
3. The dry aggregate is then immersed in heated asphalt, which is maintained at a temperature of 130 °C~150 °C for a period of 45 s. This ensures that the asphalt is completely coated with the aggregate.
4. As shown in Figure 1a, the asphalt-coated aggregate is then lifted and hung on the prepared test frame, allowing the excess asphalt to drip freely. It is then cooled at room temperature for a period of more than 15 min. A beaker of water is prepared and placed on the asbestos mesh of the heating oven. The water in the beaker is boiled using the heating oven, but the water is not allowed to boil and foam. Instead, the temperature is controlled to a state of micro-boiling.
5. As shown in Figure 1b, the cooled aggregate particles covered with asphalt are lifted one by one and immersed in boiling water. Then we observed the asphalt film on the surface of the sample during the immersion process, which lasted for three minutes.

In this paper, a long-time boiling method is added to the normal boiling method test.

#### 2.2.2. Cleavage Test

**Figure 2:**
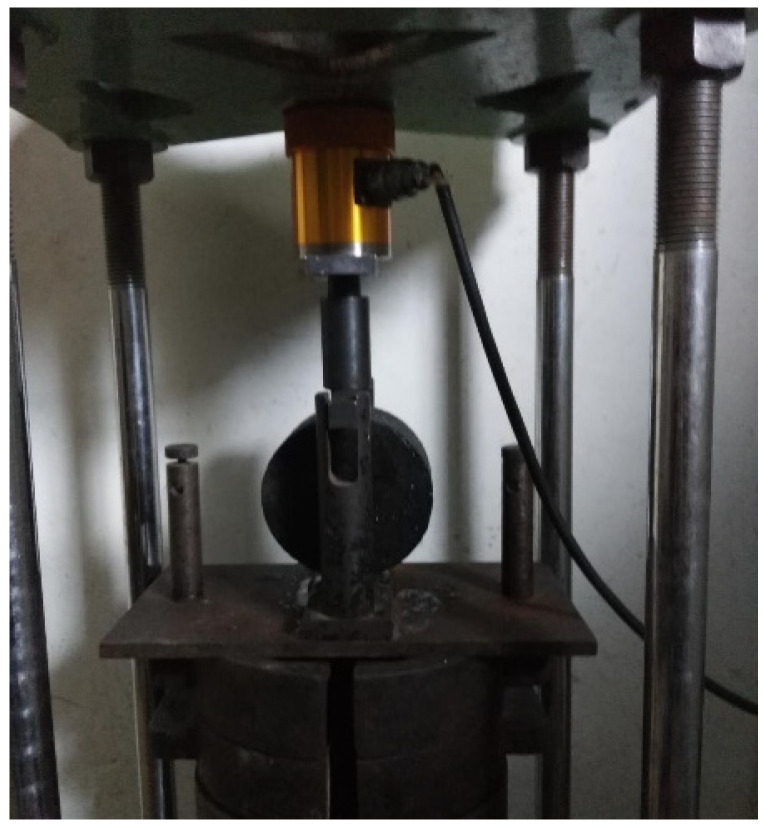
Sketch of cleavage test.

The asphalt concrete specimens doped with different contents of anti-spalling agents were tested according to the asphalt concrete test in the Test Procedure for Hydraulic Asphalt Concrete (DL/T 5362-2018). Figure 2 presents a process diagram of the splitting test. The study was conducted three times for each set of experiments, with the results averaged. If the three sets of experimental data values differed by more than 15 percent, the study was repeated. The procedure for conducting a split test is as follows:

1. The standard specimen for the splitting test was molded by the Marshall method and compacted 35 times on both sides. The diameter of the specimen mold was 101.6 mm, and the thickness of the specimen was controlled at 63.5 ± 0.5 mm. After making the specimen, the density of each specimen was measured, and the porosity was calculated. The test was carried out after 24 h of stabilization. The loading rate of the splitting test was 1.0 mm/min.
2. Test temperature: 5 ± 0.5 °C.
3. Test loading rate: 1.0 mm/min.

The main influencing factors of the mechanical properties of asphalt concrete include the nature of the raw materials, the nature of the asphalt mixtures, and the construction process. According to the test specification of asphalt and asphalt mixtures for highway engineering, the splitting tensile strength of asphalt concrete specimen (*R*_T_) was calculated according to Formula (2), the destructive tensile strain (*ε_T_*) was calculated according to Formula (3), and the modulus of strength (*S*_T_) was calculated according to Formula (4), and the performance indexes of its splitting strength, splitting displacement, and modulus of strength can be analyzed through the formula for the split strength and deformation capacity of hydraulic asphalt concrete. The strength and deformation capacity of hydraulic asphalt concrete can be analyzed, so the splitting test was an important method to study the tensile properties of asphalt concrete.

(3) $$R_{T}=0.006287P_{T}/h$$

(4) $$\varepsilon_{T}=X_{T}\times (0.0307+0.0936\mu )/\left(1.35+5\mu \right)$$

(5) ST=PT0.27+1.0μhXT

where *R_T_* is splitting tensile strength, *ε_T_* is the destructive tensile strain, *h* is the height of the specimen, *μ* is Poisson’s ratio, *X_T_* is total deformation in the horizontal direction, P_T_ is the maximum value of the specimen load, *S_T_* is the modulus of strength, *R*_t_ is the stress.

Splitting tests were carried out on asphalt concrete with parameters such as the gradation index, oil/stone ratio, and filler dosage (see Table 3, Table 4, Table 5 and Table 6 for details) derived from previous tests [37]. The proportion is shown in Table 2.

## 3. Results and Discussion

### 3.1. Results of the Boiling Method

The adhesion between asphalt doped with different contents of anti-stripping agents and gneissic amphibolite aggregate was tested according to the coarse aggregate and asphalt adhesion test (boiled water method) in the Test Procedure for Hydraulic Asphalt Concrete (DL/T 5362-2018).

**(1)**  **Asphalt adhesion test**

**Figure 3:**
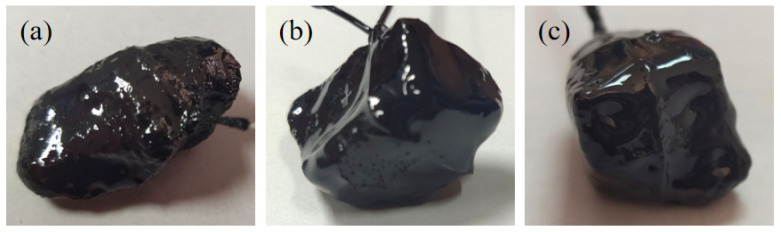
Adhesion test between asphalt with different content of anti-stripping agent and gneissic amphibolite aggregate. (**a**) No anti-peeling agent, (**b**) 0.6% anti-peeling agent, (**c**) 0.8% anti-peeling agent.

In accordance with the protocol, we tested the adhesion between asphalt (before film oven) and gneiss aggregate with different levels (Figure 3) of SK-A anti-stripping agent, and the detailed experimental data are shown in Tables S6–S13.

**Figure 4:**
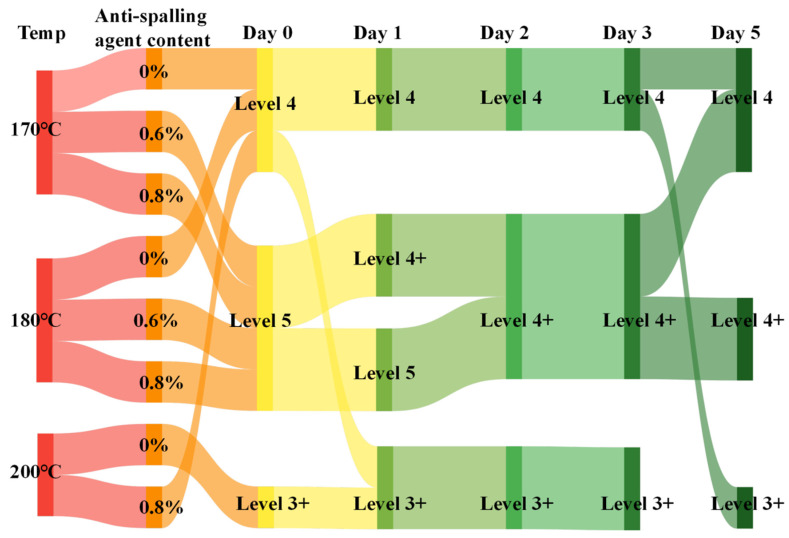
Adhesion test of asphalt to gneissic amphibolite aggregate.

According to the asphalt and aggregate adhesion test results (Figure 4), it can be seen that, compared with the anti-peeling agent dosing of 0%, the temperature increased from 170 °C to 200 °C, and the asphalt adhesion changed from level 4 to 3+ level. When the temperature was fixed, the anti-peeling agent dosing increased from 0 to 0.8, and the asphalt adhesion increased from level 4 to 5. When the temperature and the anti-peeling agent dosing were fixed, with the increase in the duration of the static time from 0 to 5 days, there was a different degree of reduction in the asphalt adhesion trend days, and the asphalt adhesion tended to decrease in different degrees.

According to the experimental results, the temperature, resting time, anti-stripping agent dosage, and other resting conditions had certain effects on asphalt adhesion; however, the influence of asphalt adhesion has a certain constraining effect on the mutual influence between storage conditions. For this purpose, temperature changes, the residence time, and the anti-stripping agent dosage were analyzed and discussed one by one.

**(2)**  **Study of the effect of resting time**

**Figure 5:**
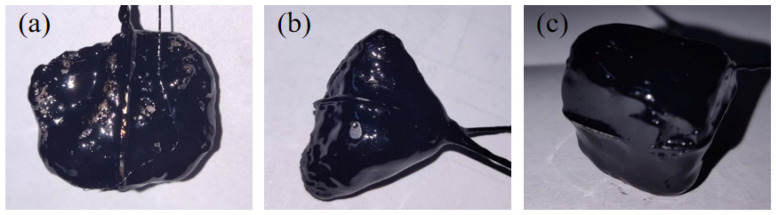
Adhesion test between asphalt with different content of anti-stripping agent and gneissic amphibolite aggregate: (**a**) no anti-stripping agent, (**b**) 0.6% anti-stripping agent, (**c**) 0.8% anti-stripping agent.

The adhesion between asphalt and gneiss aggregate after the film oven and mixing with different contents (0%, 0.6%, 0.8%) of SK-A anti-stripping agent was tested according to the test protocol; some specimens are shown in Figure 5. As the amount of anti-stripping agent increased, the coarse aggregate coated with asphalt on the surface exhibited smoother and more uniform characteristics, without exposed particles or voids. The outer surface of the coarse aggregate appeared to be more viscous, and the surface of the coarse aggregate may have taken on a darker color or even a shiny character. The test results are shown in Tables S14–S16.

**Figure 6:**
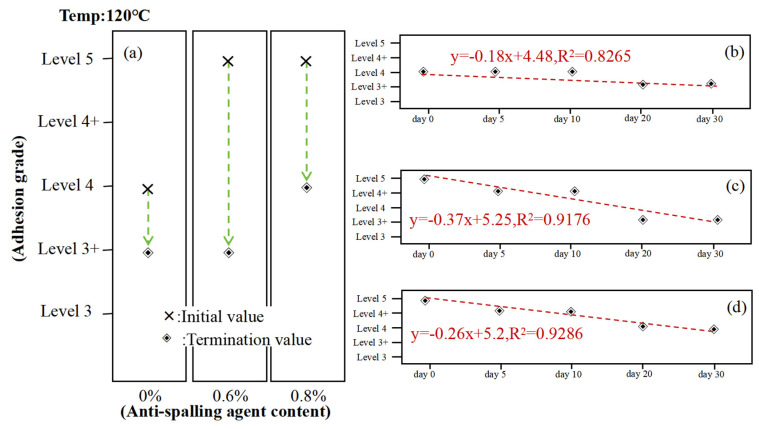
Long-term constant temperature asphalt adhesion test with gneissic amphibolite aggregate: (**a**) asphalt adhesion rating chart, (**b**) 0% anti-stripping resist bitumen adhesion rating chart, (**c**) 0.4% anti-stripping resist bitumen adhesion rating chart, (**d**) 0.8% anti-stripping resist bitumen adhesion rating chart.

Figure 6 illustrates the adhesion of asphalt to acidic coarse aggregate, employing the pertinent literature for the specific method [38]. The same methodology is employed in the following figure. From the test results, it can be seen that the use of CWS SK-A anti-stripping agent can significantly improve the adhesion of asphalt to the gneissic amphibolite aggregate. However, after 30 days of constant temperature resting, the adhesion of asphalt doped with anti-stripping agent decreases from 5 to 4, while the adhesion of asphalt without anti-stripping agent doped decreases from 4 to 3+, which indicates that the resting time is a condition that influences the adhesion of asphalt and further confirmed that the anti-stripping agent doping cannot change the end of the adhesion of asphalt with the resting time.

**(3)**  **Temperature impact studies**

**Figure 7:**
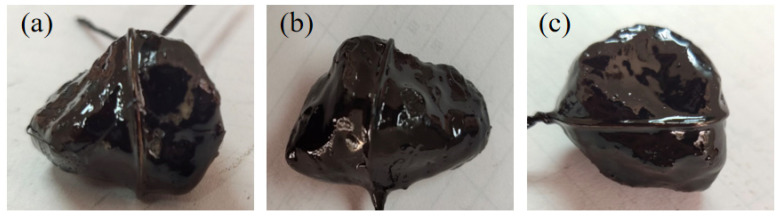
Adhesion test between asphalt with different content of anti-stripping agent and gneissic amphibolite aggregate: (**a**) no anti-stripping agent, (**b**) 0.6% anti-stripping agent, (**c**) 0.8% anti-stripping agent.

In order to investigate the effect of temperature on the adhesion between asphalt doped with SK-A anti-spalling agent and gneiss aggregate, three types of samples were prepared as shown in Figure 7. The adhesion between asphalt and gneiss aggregate doped with varying anti-spalling agent contents (0%, 0.6%, and 0.8%) was evaluated at temperatures of 140 °C, 150 °C, 160 °C, 170 °C, and 180 °C. The test results are shown in Tables S17–S19.

**Figure 8:**
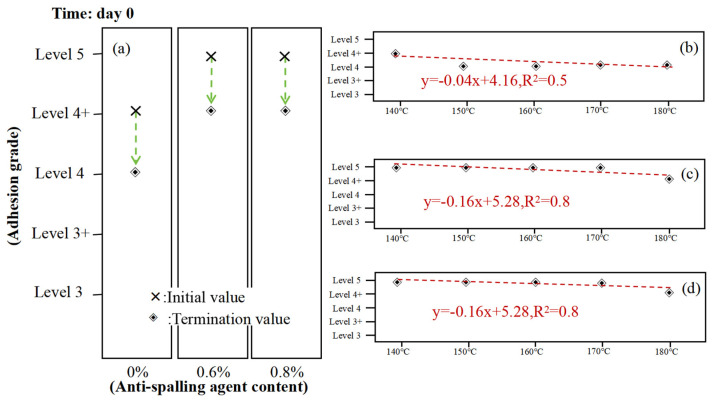
Adhesion test between asphalt and gneissic amphibolite aggregate at different temperatures: (**a**) asphalt adhesion rating chart, (**b**) 0% anti-stripping resist bitumen adhesion rating chart, (**c**) 0.6% anti-stripping resist bitumen adhesion rating chart, (**d**) 0.8% anti-stripping resist bitumen adhesion rating chart.

As can be seen from Figure 8, the asphalt adhesion tended to decrease as the temperature increased. However, the influence of temperature was not too large for the changing trend of the effect of the resting time on the asphalt adhesion; after the addition of the anti-stripping agent, its role may be minimal and still cannot change the results of the asphalt adhesion with the change in temperature.

**(4)**  **Study of the effect of anti-scaling agents**

In order to exclude the influence of the type of anti-stripping agent on this test, a new generation of non-amine asphalt anti-stripping agent (SA-100, Jiangsu Subotek, Suzhou, China) and SK-A-type anti-stripping agent were used to carry out a comparative test on the adhesion of asphalt and acidic aggregate. The relevant parameters are shown in Table S20.

**Figure 9:**
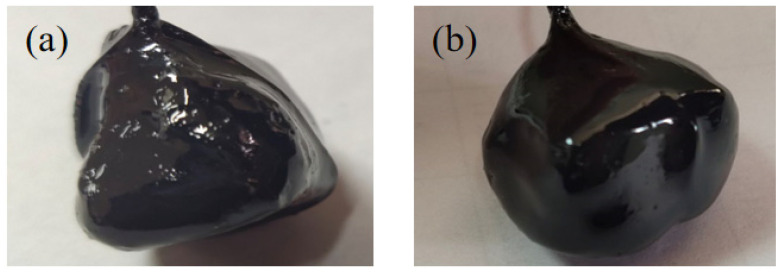
Adhesion test between asphalt with different contents of anti-stripping agent and gneissic amphibolite aggregate: (**a**) 0.4% anti-stripping agent, (**b**) 0.8% anti-stripping agent.

**Figure 10:**
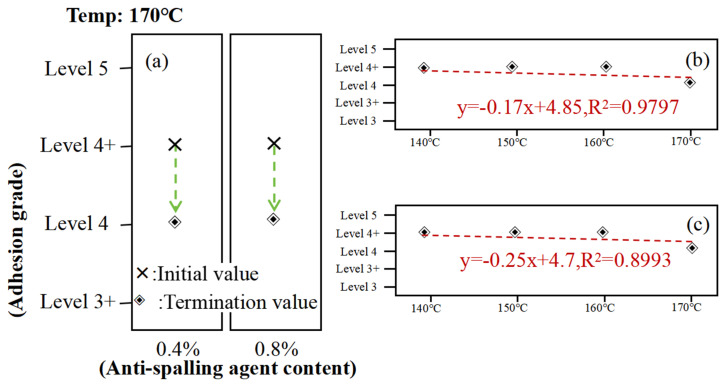
Adhesion test of different types of anti-stripping agent-resistant asphalt with gneissic amphibolite aggregate: (**a**) asphalt adhesion rating chart, (**b**) 0.4% anti-stripping resist bitumen adhesion rating chart, (**c**) 0.8% anti-stripping resist bitumen adhesion rating chart.

According to the product specification of this anti-stripping agent, the adhesion between asphalt doped with 0.4% Run strong SA-100 anti-stripping agent and gneissic amphibolite aggregate was firstly tested for 0 days, 1 day, 2 days, and 3 days at a constant temperature of 170 °C. The test samples are presented in Figure 9. As can be seen in Figure 10, when other conditions remain unchanged, the selection of SA-100 anti-stripping agent and the dosages of 0.4% and 0.8% asphalt and gneiss aggregate adhesion were reduced with the increase in the length of time of rest (Figure 10), but the rate of reduction was not large, which indicates that the type and content of anti-stripping agent did not change the asphalt adhesion to reduce the factors. The detailed results of the experiment are shown in Tables S21 and S22.

At 170 °C and 180 °C short-term constant temperatures, the adhesion levels of asphalt with gneissic amphibole aggregate decreased from 5 to 4+, 5 to 4, and 4+ to 4 for asphalt doped with 0.8% versus 0.6% anti-stripping agent and no anti-stripping agent (SK-A-type anti-stripping agent), respectively. At a long-term constant temperature of 120 °C, the SK-A type anti-scalping agent with a 0.8% dosage had the best effect. However, with the prolongation of the asphalt’s constant temperature, the asphalt gradually aged and the performance deteriorated, and the adhesion levels of the asphalt with gneissic amphibole aggregate with 0.8% versus 0.6% of the anti-stripping agent and without anti-stripping agent (SK-A-type anti-stripping agent) decreased from level 5 to level 4, level 5 to level 3+, and level 4 to level 3+, respectively. However, the aging was slower at a temperature of 120 °C. If the asphalt was blended with an SK-A-type anti-stripping agent, it was favorable for the adhesion of asphalt to acidic aggregate.

By comparing different temperature conditions from Tables S12–S17, it can be seen that, at 140 °C, 150 °C, 160 °C, 170 °C, 180 °C, and 200 °C, the adhesion between asphalt and acidic aggregate decreases from level 4 to level 3+ as the temperature increases, with a certain overall decreasing trend, but the decrease in adhesion between 140 °C and 180 °C was not significant.

The boiling method was used to study the adhesion of asphalt under different static conditions, and it can be seen that the static length and temperature influence a factor of the adhesion of asphalt, which had certain effects, and the static-length factor influence was more significant. The type of anti-stripping agent and the amount of mixing have almost no effect on the change in asphalt adhesion and the two different anti-stripping agents for the adhesion of asphalt and coarse aggregate in the different static length adhesions. After the test, there was a certain decrease in the asphalt concrete adhesion properties; the nature of the decline in adhesion was a reduction in the bond between the asphalt and aggregate, which in turn affected its mechanical properties. Therefore, it was necessary to carry out research on the effect of acidic aggregate hydraulic asphalt concrete on the mechanical properties.

### 3.2. Cleavage Test Results

**Figure 11:**
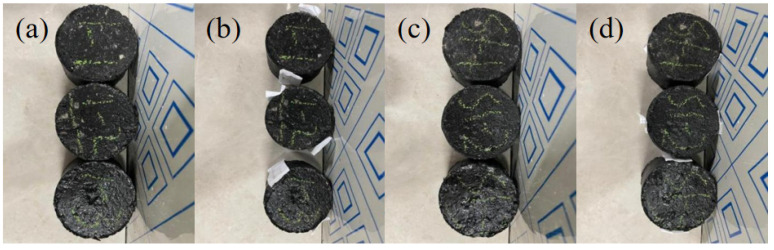
Splitting specimens: (**a**) specimen before splitting test, (**b**) specimen after splitting test, (**c**) specimen before splitting test (SK-A), (**d**) specimen after splitting test (SK-A).

Based on the study of the effect of the boiling method on the adhesion of asphalt to acidic aggregate under different resting conditions, it can be seen that the influencing factor of the resting time length was dominant. Therefore, in order to exclude the interference of the temperature and anti-stripping agent influencing factors, this chapter determines the initial factor temperature as 160 °C and the anti-stripping agent dosage as 0.6%, based on the splitting test for the mechanical properties of asphalt concrete under different resting time lengths, and it analyzes in detail the time-varying characteristics of the tensile properties after resting time lengths of 0, 10, 20, and 30 days, and the calculation results are shown in Table 8. The results of the test samples are presented in Figure 11.

**Figure 12:**
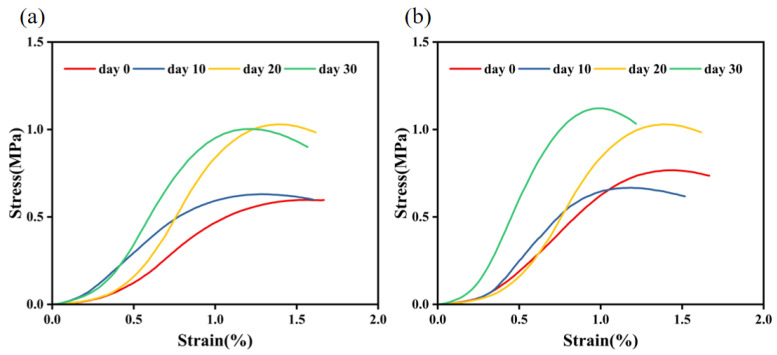
Splitting test diagrams: (**a**) stress–strain diagram for the splitting test without an anti-stripping agent, (**b**) stress–strain diagram for splitting test with an anti-stripping agent.

From the stress–strain curve of the splitting test (Figure 12), it can be seen that, whether the anti-stripping agent was added or not, with the increase in the resting time, the modulus of strength also increased gradually; the splitting strength tended to increase, and the split displacement was negatively correlated with resting time duration. After 30 days of resting, the aging of asphalt led to a decrease in the adhesion between asphalt and aggregate, which ultimately manifested itself in a decrease in the bond of the asphalt mixture [39,40,41]. The final manifestation was the reduction in the bonding force of the asphalt mixture. When the bond of the asphalt mixture decreased, the overall ability to be subjected to external forces also decreased. Therefore, the aging asphalt mixture was more prone to the phenomenon of asphalt anti-stripping under the action of load and water, which further accelerated the water damage of the asphalt concrete. In addition, asphalt aging also led to the decline of the overall structural performance of water conservancy projects and even the occurrence of functional damage to the impermeable structure.

Under the condition of 160 °C, two kinds of asphalt were subjected to tensile and splitting tests after 0, 10, 20, and 30 days of constant temperature resting, and the test results found that, after 30 days of resting, the strain of the splitting specimen was obviously decreased, and the splitting specimen’s splitting displacement, with the addition of SK-A-type anti-stripping agent, was decreased from 1.494 mm to 1.025 mm. The splitting displacement without the addition of an anti-peeling agent was decreased from 1.695 mm to 1.234 mm. The splitting displacement of both asphalt types decreased after resting.

**Figure 13:**
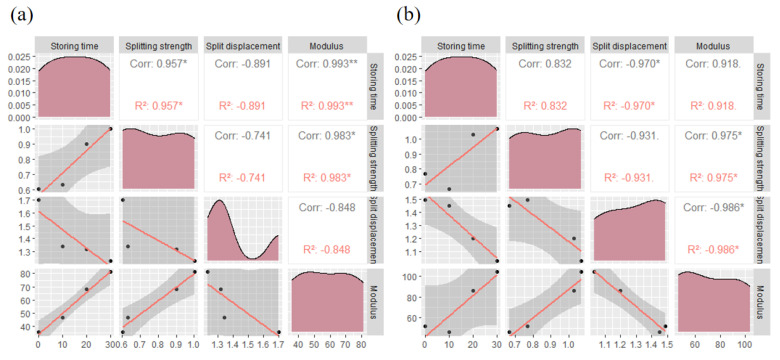
Graphs of cleavage test data: (**a**) without an anti-stripping agent; (**b**) with an anti-stripping agent. (The symbol R^2^ represents the correlation coefficient. * usually indicates that the *p*-value lies between 0.05 and 0.01, indicating statistical significance, but not very high significance. ** probability value of p less than 0.01 indicates high statistical significance).

A close relationship also existed between the splitting strength, splitting displacement, and the modulus of strength of asphalt concrete (Figure 13). Asphalt concrete with a higher modulus of strength also had a higher splitting strength. This is due to the fact that concrete with a higher modulus of strength was more rigid when subjected to stresses and can withstand greater stresses, thus requiring greater stresses to initiate rupture during splitting. Conversely, splitting displacement was negatively correlated with the modulus of strength, with concrete with a higher modulus of strength typically experiencing less displacement prior to splitting, whereas concrete with a low modulus of strength was relatively soft and more prone to larger displacements. Higher splitting strengths were accompanied by smaller splitting displacements due to the higher stresses that need to be overcome in order to initiate splitting in splitting tests, which resulted in smaller displacements.

According to the test results, asphalt aging led to a decrease in the cracking resistance and stability of asphalt concrete, which was prone to problems such as cracking and water damage. This was mainly due to the following two reasons: (1) the asphalt became hard and tough after aging, and was more likely to crack under the same temperature conditions; (2) as the asphalt’s ability to adapt to deformation decreases, it becomes more brittle and more sensitive to loads, and its stress relaxation capacity decreases. A good stress relaxation capacity prevents asphalt mixtures from cracking during temperature changes.

## 4. Conclusions

Through the indoor test, from the adhesion force and tensile property change law of asphalt aggregate and asphalt concrete, we focused on the asphalt’s different resting time lengths and temperature with respect to the acidic aggregate hydraulic asphalt concrete tensile performance to carry out research, and the main research results were as follows:

1. In the short-term constant temperature tests at 170 °C and 180 °C, the adhesion levels of the asphalt and aggregate decreased from level 5 to 4+, 5 to 4, and 4+ to 4 when the additive amount of the anti-stripping agent (SK-A anti-stripping agent) was 0.8%, 0.6%, and 0%, respectively. In the 120 °C long-term constant temperature test, with the extension of the asphalt’s constant temperature time and the asphalt gradually aging, mixed with 0.8% and 0.6% anti-stripping agent and unadulterated anti-stripping agent (SK-A-type anti-stripping agent), the asphalt and gneiss aggregate adhesion level fell from 5 to 4, 5 to 3+, 4 to 3+, respectively. That is, whether anti-stripping agent was added or not, with the temperature increased and time extended when in the static state, the adhesion of asphalt and acidic aggregate was presented in varying degrees of decline, and the addition of anti-stripping agent can slow down the degree of attenuation but cannot change the results of the reduction in adhesion.
2. Among several different resting conditions, such as the temperature, resting time, anti-stripping agent type, and dosage, the resting time exhibited the most obvious effect on asphalt adhesion.
3. The mechanical properties of the two kinds of asphalt were investigated by a splitting test with respect to the factor of resting time, and it was found that the splitting strength of splitting specimens without an anti-stripping agent increased from 0.604 MPa to 0.630 MPa, 0.904 MPa, and 1.004 MPa, respectively, while the splitting displacement decreased from 1.70 mm to 1.34 mm, 1.32 mm, and 1.23 mm, respectively. The modulus of strength increased from 35 MPa to 83.31 MPa after resting for 10, 20, and 30 days, respectively. the splitting displacement of the specimen doped with an anti-stripping agent decreased from 1.49 mm to 1.45 mm, 1.20 mm, and 1.03 mm, with decrease rates of 3%, 20%, and 31%, which reflect the deterioration of asphalt concrete and the degradation of structural properties. It was deduced that doping with an anti-stripping agent could not change the tensile properties of asphalt concrete, which decreased with the increase in the resting time.

Despite the promising results yielded by this study, there are still several areas that require further investigation.

1. Despite the comprehensive insight gained into the factors influencing asphalt adhesion properties, further research is required to ascertain the long-term performance alterations under varying environmental circumstances and material variables. Furthermore, it would be beneficial to investigate more effective asphalt modification methods and the application of anti-stripping agents to improve the durability of asphalt concrete.
2. Currently, the water-boiling method represents the principal experimental approach for evaluating the adhesion of asphalt to aggregates. Although the area ratio was employed in this study for the assessment of adhesion, this approach may also result in significant experimental errors. Consequently, it is recommended that a more objective and precise quantitative analysis method be employed in subsequent studies to enhance the accuracy and reliability of the experimental results.
3. Research should be conducted to investigate the effects of extreme climatic conditions (e.g., extreme cold, extreme heat, high humidity, etc.) on the performance of asphalt concrete, with a view to proposing corresponding design and construction strategies.

## Figures and Tables

**Table 1:** Jingbo Petrochemical-modified I-C asphalt test results table.

Serial Number	Identification of Items		Unit (Of Measure)	Technical Indicators	Test Results
1	Needle penetration (25 °C, 100 g, 5 s)		1/10 mm	60–80	74
2	Needle penetration index PI		°C	≥−0.4	4.22
3	Elongation (5 cm/min, 15 °C)		cm	/	85.67
4	Elongation (5 cm/min, 5 °C)		cm	≥30	36
5	Softening point (global method)		°C	≥55	71.56
6	Solubility (trichloroethylene)		%	≥99	99.78
7	Crispy point		°C	≤−10	−20
8	Storage stability 48 h softening point Difference after segregation		/	≤2.5	0.4
9	Flash point (open method)		°C	≥230	288
10	Density (25 °C)		g/cm	on-the-spot survey	1.029
11	Wax content (cracking method)		%	≤2.0	0.9
12	Kinematic viscosity at 135 °C		pa.s	≤3	1.3
13	Elastic recovery 25 °C		%	≥65	95
14	After film oven	Mass loss	%	≤1.0	−0.108
		Needle penetration ratio (25 °C)	%	≥60	71.8
		Elongation (15 °C, 5 cm/min)	cm	≥80	64.17
		Elongation (5 °C, 5 cm/min)	cm	≥20	18
		Elevated softening point	°C	≤5	0.55

**Table 2:** Test results of Jingbo Petrochemical-modified I-C asphalt doped with 0.8% SK-A anti-stripping agent.

Serial Number	Identification of Items		Unit (Of Measure)	Technical Indicators	Test Results
1	Needle penetration (25 °C, 100 g, 5 s)		1/10 mm	60–80	75.67
2	Needle penetration index PI		°C	≥−0.4	4.2
3	Elongation (5 cm/min, 15 °C)		cm	/	79
4	Elongation (5 cm/min, 5 °C)		cm	≥30	35
5	Softening point (global method)		°C	≥55	70.1
6	Solubility (trichloroethylene)		%	≥99	99.77
7	Crispy point		°C	≤−10	−20
8	Storage stability 48 h softening point Difference after segregation		/	≤2.5	0.4
9	Flash point (open method)		°C	≥230	287
10	Density (25 °C)		g/cm	on-the-spot survey	1.03
11	Wax content (cracking method)		%	≤2.0	0.9
12	Kinematic viscosity at 135 °C		pa.s	≤3	1.3
13	Elastic recovery 25 °C		%	≥65	95
14	After film oven	Mass loss	%	≤1.0	−0.144
		Needle penetration ratio (25 °C)	%	≥60	89.54
		Elongation (15 °C, 5 cm/min)	cm	≥80	73
		Elongation (5 °C, 5 cm/min)	cm	≥20	17
		Elevated softening point	°C	≤5	0.6

**Table 3:** Gneiss diorite test report.

Room Number	Commission Number	ω_B_/10^−2^						
		SiO_2_	Al_2_O_3_	Fe_2_O_3_	MgO	CaO	Heat Loss	Alkalinity Modulus (Chemistry)
**23D161-1**	1#	72.25	4.95	3.52	2.29	6.84	2.17	0.18

**Table 4:** Coarse aggregate selection criteria table.

Serial Number	Sports Event	Unit (of Measure)	Design Requirements	Test Results
1	Apparent density	g/cm^3^	≥2.6	2.72
2	Adhesion to asphalt	classifier: step, level	≥4	4
3	Content of needle and flake particles	%	≤25	6.97
4	Crushing value	%	≤30	9.56
5	Water absorption	%	≤2	0.43
6	Mud content	%	≤0.5	0
7	Durability (Sodium sulfate mass loss from 5 wet and dry cycles)	%	≤12	4.2

Note: The requirement indexes are derived from the requirement indexes for asphalt concrete coarse aggregate in the water conservancy industry standard of the People’s Republic of China, Design Code for Asphalt Concrete Panels and Heart Walls of Earth and Stone Dams [34].

**Table 5:** Fine aggregate material selection table.

Serial Number	Sports Event	Unit (of Measure)	Required Indicators	Test Results
1	Apparent Density	g/cm^3^	≥2.55	2.71
2	Water Absorption	%	≤2	-
3	Water Stability Rating *	classifier: step, level	≥6	6
4	Durability (Sodium sulfate mass loss from 5 wet and dry cycles)	%	≤15	1.2
5	Stone Powder Content *	%	<5	

Note: (1) The requirement indexes are from the requirement indexes for asphalt concrete fine aggregate in the water conservancy industry standard of the People’s Republic of China, Design Code for Asphalt Concrete Panels and Heart Walls of Soil and Stone Dams (DL/T 5411-2009), except those with *. (2) The asphalt in the water-stabilized grades is asphalt modified by Kyobo Petrochemicals without the addition of anti-stripping agents.

**Table 6:** Filler selection criteria table.

Serial Number	Sports Event		Unit (of Measure)	Required Indicators	Test Results
1	Apparent Density		g/cm^3^	≥2.5	2.715
2	Moisture Content		%	≤0.5	---
3	Hydrophilicity		---	≤1.0	0.628
4	Thin and soft degree	<0.6 mm	%	100	100
		<0.15 mm		>90	99.7
		<0.075 mm		>85	86.5

Note: The requirement indexes are derived from the requirement indexes for asphalt concrete filler in the water conservancy industry standard of the People’s Republic of China, Design Code for Asphalt Concrete Panels and Heart Walls of Earth and Stone Dams (DL/T 5411-2009).

**Table 7:** Preferred mix ratio parameter table.

Greatest Grain Size	Gradation Exponents	Filler Amount F	Oil/Stone Ratio B	Percentage of Mass of Each Level of Mineral (%)					
mm	r	**%**	**%**	19–13.2 (mm)	13.2–9.5 (mm)	9.5–4.75 (mm)	4.75–2.36 (mm)	2.36–0.075 (mm)	<0.075 (mm)
19	0.5	9	7	16.17	12.27	20.11	14.33	28.12	9

**Table 8:** Table of asphalt concrete splitting test results.

Specimen Type	Density (g/cm)^3^	Porosity (%)	Maximum Splitting Strength (MPa)	Maximum Splitting Displacement(mm)	Modulus of Strength (MPa)
No anti-stripping agent	2.418	0.77	0.604	1.70	35.53
	2.412	1.06	0.630	1.34	47.02
	2.406	1.31	0.901	1.32	68.26
	2.419	0.78	1.004	1.23	81.23
Add SK-A anti-stripping agent	2.425	0.53	0.769	1.49	51.61
	2.417	0.86	0.668	1.45	46.07
	2.419	0.78	1.030	1.20	85.83
	2.411	1.11	1.071	1.03	103.98

## Data Availability

Data are contained within the article and Supplementary Materials.

## Supplementary Materials

The following supporting information can be downloaded at: https://www.mdpi.com/article/10.3390/ma17143556/s1, Table S1: Jingbo Petrochemical modified I-C asphalt test results table; Table S2: Test results of Jingbo Petrochemical modified I-C asphalt doped with 0.8% SK-A anti-stripping agent; Table S3: Coarse aggregate selection criteria table; Table S4: Fine Aggregate Material Selection Table; Table S5: Filler selection criteria table; Table S6: Results of 170 °C constant temperature test of asphalt without spalling agent; Table S7: Addition of 0.6% SK-A anti-spalling agent asphalt 170 °C constant temperature test results table; Table S8: Addition of 0.8% SK-A anti-stripping agent asphalt 170 °C constant temperature test results table; Table S9: Results of 180 °C constant temperature test of asphalt without spalling agent; Table S10: Addition of 0.6% SK-A anti-stripping agent asphalt 180 °C constant temperature test results table; Table S11: Addition of 0.8% SK-A anti-stripping agent asphalt 180 °C constant temperature test results table; Table S12: Results of 200 °C constant temperature test of asphalt without spalling agent; Table S13: Addition of 0.8% SK-A anti-stripping agent asphalt 200 °C constant temperature test results table; Table S14: Results of 120 °C constant temperature test of asphalt without spalling agent; Table S15: Addition of 0.6% SK-A anti-stripping agent asphalt 120 °C constant temperature test results table; Table S16: Addition of 0.8% SK-A anti-spalling agent asphalt 120 °C constant temperature test results table; Table S17: Results of the temperature dependence test of adhesion without spalling inhibitors; Table S18: Addition of 0.6% SK-A anti-scaling agent adhesion with temperature test results table; Table S19: Addition of 0.8% SK-A anti-spalling agent adhesion with temperature change test results table; Table S20: Technical parameters of Runqiang-SA100 asphalt anti-spalling agent; Table S21: Adhesion test results of 0.4 per cent Runqiang SA-100 anti-scalping agent added; Table S22: Adhesion test results of 0.8 per cent Runqiang SA-100 anti-scalping agent added.
