# Supplementary material for: Effects of Resting Conditions on Tensile Properties of Acid Aggregate Hydraulic Asphalt Concrete
Source: Materials (Basel). 2024 Jul 18;17(14):3556. doi: 10.3390/ma17143556 (PMC11278492; doi:10.3390/ma17143556)
Supplement: Supplementary file 1 [file materials-17-03556-s001.zip › materials-3010049-supplementary.pdf]

materials

Supplementary Information

Effects of Resting Conditions on Tensile Properties of Acid Aggregate Hydraulic Asphalt Concrete

Lei Bao [1,2], Min He [1,3,*], Shu Wang [2] and Xinshuang Wu [2]

[1] School of Civil Engineering and Architecture, Xi'an University of Technology, Xi'an 710048, China; lbao1013@163.com
[2] Power China Northwest Engineering Corporation Limited, Xi'an 710065, China; 13102213129@163.com (S.W.); wuxins@nwh.cn (X.W.)
[3] State Key Laboratory of Eco-Hydraulics in Northwest Arid Region of China, Xi'an University of Technology, Xi'an 710048, China
* Correspondence: hem@xaut.edu.cn; Tel.: +86-13309201657

Table S1. Jingbo Petrochemical modified I-C asphalt test results table.

Serial number	Identification of items		unit (of measure)	Technical indicators	Test results
1	Needle penetration (25°C, 100g, 5s)		1/10mm	60-80	74
2	Needle penetration index PI		°C	≥-0.4	4.22
3	Elongation (5 cm/min, 15°C)		cm	/	85.67
4	Elongation (5 cm/min, 5°C)		cm	≥30	36
5	Softening point (global method)		°C	≥55	71.56
6	Solubility (trichloroethylene)		%	≥99	99.78
7	crispy point		°C	≤-10	-20
8	Storage stability 48h softening point difference after segregation		/	≤2.5	0.4
9	Flash point (open method)		°C	≥230	288
10	Density (25°C)		g/cm	on-the-spot survey	1.029
11	Wax content (cracking method)		%	≤2.0	0.9
12	Kinematic viscosity at 135°C		pa.s	≤3	1.3
13	Elastic recovery 25°C		%	≥65	95
14	After film oven	mass loss	%	≤1.0	-0.108
		Needle penetration ratio (25°C)	%	≥60	71.8
		Elongation (15°C, 5 cm/min)	cm	≥80	64.17
		Elongation (5°C, 5 cm/min)	cm	≥20	18
		Elevated softening point	°C	≤5	0.55

Note:The testing indexes are from the national standard "Testing Procedure for Hydraulic Asphalt Concrete" (DL/T 5362-2018).

Table S2. Test results of Jingbo Petrochemical modified I-C asphalt doped with 0.8% SK-A anti-stripping agent.

Serial number	Identification of items		unit (of measure)	Technical indicators	Test results
1	Needle penetration (25℃, 100g, 5s)		1/10mm	60-80	75.67
2	Needle penetration index PI		℃	≥-0.4	4.2
3	Elongation (5 cm/min, 15℃)		cm	/	79
4	Elongation (5 cm/min, 5℃)		cm	≥30	35
5	Softening point (global method)		℃	≥55	70.1
6	Solubility (trichloroethylene)		%	≥99	99.77
7	crispy point		℃	≤-10	-20
8	Storage stability 48h softening point difference after segregation		/	≤2.5	0.4
9	Flash point (open method)		℃	≥230	287
10	Density (25℃)		g/cm	on-the-spot survey	1.03
11	Wax content (cracking method)		%	≤2.0	0.9
12	Kinematic viscosity at 135℃		pa.s	≤3	1.3
13	Elastic recovery 25℃		%	≥65	95
14	After film oven	mass loss	%	≤1.0	-0.144
		Needle penetration ratio (25℃)	%	≥60	89.54
		Elongation (15℃, 5 cm/min)	cm	≥80	73
		Elongation (5℃, 5 cm/min)	cm	≥20	17
		Elevated softening point	℃	≤5	0.6

Note:The testing indexes are from the national standard "Testing Procedure for Hydraulic Asphalt Concrete" (DL/T 5362-2018).

Table S3. Coarse aggregate selection criteria table.

Serial number	sports event	unit (of measure)	Design Requirements	Test results
1	apparent density	g/cm^3	≥2.6	2.72
2	Adhesion to asphalt	classifier: step, level	≥4	4
3	Content of needle and flake particles	%	≤25	6.97
4	Crushing value	%	≤30	9.56
5	water absorption	%	≤2	0.43
6	mud content	%	≤0.5	0
7	durability (Sodium sulphate mass loss from 5 wet and dry cycles)	%	≤12	4.2

Note: The requirement indexes are derived from the requirement indexes for asphalt concrete coarse aggregate in the water conservancy industry standard of the People's Republic of China, Design Code for Asphalt Concrete Panels and Heart Walls of Earth and Stone Dams (DL/T 5411-2009).

Table S4. Fine Aggregate Material Selection Table.

Serial number	sports event	unit (of measure)	Required indicators	Test results
1	apparent density	g/cm³	≥2.55	2.71
2	water absorption	%	≤2	-
3	Water stability rating*	classifier: step, level	≥6	6
4	durability (Sodium sulphate mass loss from 5 wet and dry cycles)	%	≤15	1.2
5	Stone powder content*	%	<5	

Note: (1) The requirement indexes are from the requirement indexes for asphalt concrete fine aggregate in the water conservancy industry standard of the People's Republic of China, Design Code for Asphalt Concrete Panels and Heart Walls of Soil and Stone Dams (DL/T 5411-2009), except those with *. (2) The asphalt in the water-stabilised grades is Kyobo Petrochemicals modified asphalt without the addition of anti-stripping agents.

Table S5. Filler selection criteria table.

Serial number	sports event		unit (of measure)	Required indicators	Test results
1	apparent density		g/cm3	≥2.5	2.715
2	moisture content		%	≤0.5	---
3	hydrophilicity		---	≤1.0	0.628
4	thin and soft degree (angles, temperature etc)	<0.6mm	%	100	100
		<0.15mm		＞90	99.7
		<0.075mm		＞85	86.5

Note: The requirement indexes are derived from the requirement indexes for asphalt concrete filler in the water conservancy industry standard of the People's Republic of China, Design Code for Asphalt Concrete Panels and Heart Walls of Earth and Stone Dams (DL/T 5411-2009).

Table S6. Results of 170°C constant temperature test of asphalt without spalling agent.

Anti-scaling agent content	Serial number	Adhesion grade (0 days)	Adhesion grade (1 day)	Adhesion grade (2 days)	Adhesion grade (3 days)	Adhesion grade (5 days)
8th earthly branch: 1-3 p.m., 6th solar month (7th July-6th August)	1	4+	4+	4+	4	4
	2	4+	4+	4	4	4
	3	4	4	4	4	4
	4	4	4	4	4	4
grasp plus	5	4	4	4	4	4
Comprehensive rating		4	4	4	4	4

Table S7. Addition of 0.6% SK-A anti-spalling agent asphalt 170 °C constant temperature test results table.

Anti-scaling agent content	Serial number	Adhesion grade (0 days)	Adhesion grade (1 day)	Adhesion grade (2 days)	Adhesion grade (3 days)	Adhesion grade (5 days)
0.6 per cent	1	5	5	5	4+	4+
	2	5	5	5	4+	4
	3	5	4+	4+	4+	4
	4	4+	4	4	4	4
Comprehensive rating		5	4+	4+	4+	4

Table S8. Addition of 0.8% SK-A anti-stripping agent asphalt 170 °C constant temperature test results table.

Anti-scaling agent content	Serial number	Adhesion grade (0 days)	Adhesion grade (1 day)	Adhesion grade (2 days)	Adhesion grade (3 days)	Adhesion grade (5 days)
0.8 per cent	1	5	5	5	4+	4+
	2	5	5	4+	4+	4+
	3	5	5	4+	4+	4+
	4	4+	4+	4+	4	4
	5	4+	4+	4+	4+	4+
Comprehensive rating		5	5	4+	4+	4+

Table S9. Results of 180°C constant temperature test of asphalt without spalling agent.

Anti-scaling agent content	Serial number	Adhesion grade (same day)	Adhesion grade (1 day)	Adhesion grade (2 days)	Adhesion grade (3 days)	Adhesion grade (5 days)
8th earthly branch: 1-3 p.m., 6th solar month (7th July-6th August) grasp plus	1	4+	4+	4++	4	4
	2	4+	4	4+	4	3+
	3	4	4	4+	4	3+
	4	4	4	4	4	3+
unadulterated	5	4	4	4	4	3+
Comprehensive rating		4	4	4	4	3+

Table S10. Addition of 0.6% SK-A anti-stripping agent asphalt 180 °C constant temperature test results table.

Anti-scaling agent content	Serial number	Adhesion grade (same day)	Adhesion grade (1 day)	Adhesion grade (2 days)	Adhesion grade (3 days)	Adhesion grade (5 days)
0.6 per cent	1	5	5	4+	4+	4+
	2	5	4+	4+	4+	4+
	3	5	4+	4+	4+	4
	4	4+	4+	4+	4+	4
	5	4	4	4+	4	4
Comprehensive rating		5	4+	4+	4+	4

Table S11. Addition of 0.8% SK-A anti-stripping agent asphalt 180℃ constant temperature test results table.

Anti-scaling agent content	Serial number	Adhesion grade (same day)	Adhesion grade (1 day)	Adhesion grade (2 days)	Adhesion grade (3 days)	Adhesion grade (5 days)
0.8 per cent	1	5	5	4+	4+	4+
	2	5	5	4+	4+	4+
	3	5	5	4+	4+	4+
	4	5	4	4	4+	4
	5	4+	4+	4	4	4
Comprehensive rating		5	5	4+	4+	4+

Table S12. Results of 200℃ constant temperature test of asphalt without spalling agent.

Anti-scaling agent content	Serial number	Adhesion grade (same day)	Adhesion grade (1 day)	Adhesion grade (2 days)	Adhesion grade (3 days)
8th earthly branch: 1-3 p.m., 6th solar month (7th July-6th August) grasp plus	1	4	3+	3+	3+
	2	4	3+	3+	3+
	3	3+	3+	3+	3+
	4	3+	3+	3+	3+
	5	3+	3+	3+	3+
Comprehensive rating		3+	3+	3+	3+

Table S13. Addition of 0.8% SK-A anti-stripping agent asphalt 200 ℃ constant temperature test results table.

Anti-scaling agent content	Serial number	Adhesion grade (same day)	Adhesion grade (1 day)	Adhesion grade (2 days)	Adhesion grade (3 days)
0.6 per cent	1	4	4	3+	3+
	2	4	4	3+	3+
	3	4	3+	3+	3+
	4	3+	3+	3+	3+
	5	3+	3+	3+	3+
Comprehensive rating		4	3+	3+	3+

Table S14. Results of 120℃ constant temperature test of asphalt without spalling agent.

Anti-scaling agent content	Serial number	Adhesion grade (same day)	Adhesion grade (5 days)	Adhesion grade (10 days)	Adhesion grade (20 days)	Adhesion grade (30 days)
8th earthly branch: 1-3 p.m., 6th solar month (7th July-6th August) grasp plus	1	5	4+	4+	4	4
	2	4	4+	4	3+	3+
	3	4	4	4	3+	3+
	4	4	4	4	3+	3+
	5	4	4	4	3+	3+
Comprehensive rating		4	4	4	3+	3+

Table S15. Addition of 0.6% SK-A anti-stripping agent asphalt 120℃ constant temperature test results table.

Anti-scaling agent content	Serial number	Adhesion grade (same day)	Adhesion grade (5 days)	Adhesion grade (10 days)	Adhesion grade (20 days)	Adhesion grade (30 days)
0.6 per cent	1	5	4+	4+	4	4
	2	5	4+	4+	3+	3+
	3	5	4+	4+	3+	3+
	4	5	4	4	3+	3+
	5	5	4	4	3+	3
Comprehensive rating		5	4+	4+	3+	3+

Table S16. Addition of 0.8% SK-A anti-spalling agent asphalt 120 ℃ constant temperature test results table.

Anti-scaling agent content	Serial number	Adhesion grade (same day)	Adhesion grade (5 days)	Adhesion grade (9 days)	Adhesion grade (20 days)	Adhesion grade (30 days)
0.8 per cent	1	5	5	5	4	4
	2	5	5	4+	4	4
	3	5	4+	4+	4	4
	4	5	4+	4+	4	4
	5	5	4	4	4	4
Comprehensive rating		5	4+	4+	4	4

Table S17. Results of the temperature dependence test of adhesion without spalling inhibitors.

Anti-scaling agent content	Serial number	Adhesion grade (140°C)	Adhesion grade (150°C)	Adhesion grade (160°C)	Adhesion grade (170°C)	Adhesion grade (180°C)
8th earthly branch: 1-3 p.m., 6th solar month (7th July-6th August) grasp plus	1	5	4	4	4	4
	2	4+	4	4	4	4
	3	4+	4	4	4	4
	4	4+	4	4	4	4
	5	4	4	4	4	4
Comprehensive rating		4+	4	4	4	4

Table S18. Addition of 0.6% SK-A anti-scaling agent adhesion with temperature test results table.

Anti-scaling agent content	Serial number	Adhesion grade (140°C)	Adhesion grade (150°C)	Adhesion grade (160°C)	Adhesion grade (170°C)	Adhesion grade (180°C)
0.6 per cent	1	5	5	5	5	5
	2	5	5	5	5	4+
	3	5	5	5	5	4+
	4	5	5	5	4	4
	5	5	5	5	4	4
Comprehensive rating		5	5	5	5	4+

Table S19. Addition of 0.8% SK-A anti-spalling agent adhesion with temperature change test results table.

Anti-scaling agent content	Serial number	Adhesion grade (140°C)	Adhesion grade (150°C)	Adhesion grade (160°C)	Adhesion grade (170°C)	Adhesion grade (180°C)
0.8 per cent	1	5	5	5	5	5
	2	5	5	5	5	5
	3	5	5	5	5	4+
	4	4	4	4+	4+	4+
	5	4+	4	4	4	4
Comprehensive rating		5	5	5	5	4+

Table S20. Technical parameters of Runqiang-SA100 asphalt anti-spalling agent.

Sports event	General technical requirements	Test results for this product
Exterior condition	Uniform colour, no delamination, spots and other abnormalities	Black or brown viscous liquid
Odours	No significant extraneous irritating odour	No significant extraneous irritating odour
Volatiles content	<10 per cent	1.01 per cent
Adhesion	Before ageing, not less than class 4	5
	After ageing, not less than class 3	5
	Comparison before and after ageing, not greater than 1 level	0
	Before and after adding anti-flaking agent, not less than 1 grade	1-3

Table S21. Adhesion test results of 0.4 per cent Runqiang SA-100 anti-scalping agent added.

Pitch	Serial number	Adhesion grade (0 days)	Adhesion grade (1 day)	Adhesion grade (2 days)	Adhesion grade (3 days)
0.4 per cent	1	5	5	4+	4+
	2	5	4+	4+	4+
	3	4+	4+	4+	4
	4	4+	4+	4	4
	5	4+	4	4	4
Comprehensive rating		4+	4+	4+	4

Table S22. Adhesion test results of 0.8 per cent Runqiang SA-100 anti-scalping agent added.

Pitch	Serial number	Adhesion grade (0 days)	Adhesion grade (1 day)	Adhesion grade (2 days)	Adhesion grade (3 days)
0.8 per cent	1	5	5	4	4
	2	5	4	4	4
	3	4+	4	4	4
	4	4	4	3+	3+
	5	4	4	3+	3+
Comprehensive rating		4+	4	4	4
